# Perceptions of Collaboration between Traditional Bone Setters and Formal Care Providers in Cameroon: Bridging Gaps in Injury Care

**DOI:** 10.5334/aogh.5125

**Published:** 2026-08-24

**Authors:** Arole Darwin Touko, Mark T. Yost, Basile Ngassa, Sithombo Maqungo, Serge Ngekeng, Kudzai Chironga, Aminou Sadjo Salihou, Beme Saouwada Apollinaire, Christelle Maffo, Tonny Orach, Rasheedat Oke, Liza Rosenbloom, Catherine Juillard, Alain Chichom-Mefire

**Affiliations:** 1 Data Science Center for the Study of Surgery, Injury, and Equity in Africa (D-SINE Africa), University of Buea, Buea, Southwest, Cameroon; 2 Program for the Advancement of Surgical Equity, University of California Los Angeles, Los Angeles, California, USA; 3 Maroua Regional Hospital, Maroua, Far-North, Cameroon; 4 Division of Global Surgery, University of Cape Town, Cape Town, South Africa; 5 Sustainable Trauma Research, Education, and Mentorship (STREaM) Cameroon, Buea, Southwest, Cameroon

**Keywords:** task shifting, traditional bone setters, traditional healers, health care in Africa, injury, collaboration

## Abstract

*Introduction:* In Cameroon, there are not enough formal care (FC) providers to treat the high volume of injured patients. Traditional bone setters (TBS) offer widely patronized alternatives for injury treatment; however, the integration of TBS into the healthcare system remains limited. Collaboration between TBS and FC providers could expand the reach and effectiveness of injury care. We explore perceptions of Cameroonian TBS and FC providers regarding injury care and willingness to collaborate.

*Methodology:* We used snowball sampling to survey TBS and FC providers in two regions of Cameroon. Structured questionnaires were used to assess FC and TBS providers’ beliefs regarding potential collaboration. Categorical variables were analyzed with the chi-squared test while Likert scale responses were analyzed with the Kruskal-Wallis test.

*Results:* We surveyed 120 providers (*n*=58 TBS, *n*=62 FC). TBS providers were significantly older (TBS median age 51, IQR 39–60 years vs. FC 34, IQR 30–36 years; *P*<0.001), greater percentage male (TBS 72.4% vs. FC 51.6%, *P*=0.019), and had more years of practice experience (TBS median 20, IQR 11–28 years vs. FC 5, IQR 3–8 years; *P*<0.001). While the majority of TBS (93%) and FC providers (77%) indicated willingness to collaborate, they disagreed regarding specific roles. Though the TBS providers believed they could conduct post-operative follow-up care, FC providers disagreed (TBS median response “Agree” vs. FC “Disagree,” *P*<0.001). FC providers also did not believe TBS could provide appropriate injury care triage (TBS “Agree” vs. FC “Neutral,” *P*<0.001). TBS and FC providers agreed that TBS could rehabilitate injured patients, although TBS agreed more strongly (*P*<0.001).

*Conclusions:* The study demonstrates the willingness of both FC and TBS providers in Cameroon to collaborate in injury care, particularly in rehabilitation services. However, the cohorts disagree regarding the specific roles of TBS providers in injury triage and post-operative follow-up care.

## Introduction

Injury results in significant morbidity and mortality in Cameroon, accounting for nearly half of all emergency department visits [1]. Among injured patients who seek formal medical care, access to timely and appropriate treatment is often limited by a lack of specialized training and a dearth of available equipment [2]. However, a critical shortage of formal care (FC) providers especially strains the ability of the Cameroonian health system to treat injured patients, as there are only 11 qualified health providers per 10,000 population [3]. This dearth of providers becomes more apparent in rural locations since more than half of FC providers work in the three largest cities of Yaoundé, Douala, and Bafoussam [3].

Traditional healers, known throughout Cameroon and other countries in West and Central Africa as traditional bone setters (TBS), offer treatment for fractures and dislocations using methods such as manual manipulation, splints made from local materials, and herbal remedies [4–9]. These widely patronized practitioners offer alternative treatments that are often cheaper, more culturally familiar, or more readily accessible than formal care [8, 10]. TBS providers represent potential partners for health care task shifting to ensure greater patient access to injury care. Prior studies of traditional healers in Africa demonstrate that the integration of traditional and formal care can increase provider knowledge and results in improved care for patients [4, 5, 11]. Both the World Health Organization (WHO) and the government of Cameroon have sought to further develop the role of TBS within the existing health system [6, 12, 13]. In December 2024, Cameroon Law No. 2024/018 sought to categorize traditional practitioners, create a National Order of Traditional Health Practitioners, and codify collaboration between TBS and FC providers [13]. Due to the novelty of the law, no public data exists regarding the practical application of the law in Cameroon. Efforts to formalize collaboration between TBS and FC providers in other countries in sub-Saharan Africa have faced challenges. TBS practices are often informal and lack standardized medical training, which in some cases may lead to complications such as malunion, infection, or delayed referral [7, 14]. Likewise, the lack of an integrated system between FC and TBS providers can result in inappropriate care utilization, misdiagnosis of severe illness, and worsened patient health outcomes after injury [7, 14, 15].

These limitations highlight a critical need to better understand the interactions that exist between FC and TBS providers in Cameroon. Realistic and effective integration of TBS providers into the greater health care system requires understanding of the perceptions of both FC and TBS groups. For instance, prior analyses of traditional healers in Africa demonstrate a consistent willingness for collaboration with FC providers and a desire to improve the quality of health care services [9, 15, 16]. However, far less is known about the attitudes of FC providers toward such collaboration. This study explores the perceptions of Cameroonian TBS and FC providers toward the development of a model for collaborative practices. Specifically, our exploratory study sought to understand how FC and TBS providers perceive the potential collaboration and identify which particular care settings may be the most promising areas for both groups to begin collaborating on patient care.

## Methods

### Study design

This cross-sectional study used a structured questionnaire to collect data on perceptions of collaboration from TBS and FC providers. Prior discussions with providers, including clinical anecdotes, provided the basis for the content of the questionnaire. The questionnaire was developed specifically for this study rather than adapted from a previously validated instrument as no standardized tool assessing TBS and FC collaboration was available. Themes from prior studies of TBS in Africa informed domains of the questionnaire and were refined through discussion with local providers [4, 9, 15, 16]. The questionnaire was originally developed in English, translated to French, and back-translated to English to ensure accuracy. The survey was then piloted among FC and TBS providers prior to implementation to ensure clarity and relevance.

### Setting

The study was conducted in two regions of Cameroon, the Southwest (SW) and the Far-North (FN), respectively. Regional referral centers in the SW (Limbe Regional Hospital) and FN (Maroua Regional Hospital) regions contribute data to the Cameroon Trauma Registry (CTR), a registry collecting data from 10 hospitals located in 7 of 10 governmental regions in Cameroon. Additionally, prior injury-care literature and fieldwork observations by our study team noted a comparatively high reliance on TBS providers in these regions [10, 15]. These two regions differ significantly from the major urban centers of the country. The FN is the most rural part of the country, while the more developed SW is predominantly non-metropolitan with an ongoing sociopolitical conflict [3, 17]. While rural and economically disadvantaged areas have a scarcity of formal medical services, TBS usage is more common in these areas [3, 15].

### Participants and data collection

Study participants included TBS and FC providers practicing in the SW and FN regions of Cameroon. Snowball sampling was used to recruit both TBS and FC providers in the two study regions. We defined FC providers as doctors and nurses providing injury care in allopathic clinical settings, including regional hospitals, private hospitals, military hospitals, and district hospitals. We recruited FC providers who treated trauma patients (e.g., orthopedic surgeons, pediatric surgeons, general surgeons, and nurses) and were willing to participate in the study. These providers were identified through hospital departments and professional networks at selected facilities. TBS were defined as traditional injury care providers using knowledge, skills, and practices based on indigenous cultures. We identified TBS providers by community word-of-mouth referral and individual healer contacts. Trained research assistants orally administered the survey in either English, French, Pidgin, or Fulfulde depending on the respondent’s language preference. The research assistant received standardized training to ensure consistency in delivery and written recording of responses. The questionnaire included closed-ended items, primarily using Likert-scale formats, to measure attitudes and perceptions related to collaboration among the cohorts.

### Statistical methods and data analysis

Categorical variables from the surveys were analyzed using the chi-square test. We used Wilcoxon rank-sum test to assess differences in continuous demographic variables. Furthermore, we assigned numeric values to Likert scale responses (Strongly Disagree = 1, Disagree = 2, Neutral = 3, Agree = 4, and Strongly Agree = 5) for data analysis. We then used the Kruskal–Wallis test to compare median responses between TBS and FC provider groups and assess for statistical differences. We analyzed median responses, rather than mean responses, because they are more resistant to outliers and skewed distributions in the small samples. Numeric ranges for categorization of median and mean responses included: Strongly Disagree = 1–1.5, Disagree = 1.6–2.4, Neutral = 2.5–3.5, Agree = 3.6–4.4, and Strongly Agree = 4.5–5. Data analysis was performed in Stata version 16 [18]. Additionally, we used Microsoft Excel [19] to create radar plots and bar graphs to aid in data visualization.

### Ethics

The study received Institutional Review Board (IRB) approval at the University of Buea in Cameroon and the University of California, Los Angeles, in the United States. Prior to data collection, the study received administrative clearance from the Regional Delegation of Public Health in both the FN and SW Cameroonian governmental regions.

Informed consent was obtained from all participants prior to taking part in the study. Trained Cameroonian research assistants informed all prospective participants regarding the purpose, procedures, possible risks, and benefits of the study in their native language. Participants were also informed of their right to skip any survey questions that made them feel uncomfortable and their right to withdraw from the study at any time without penalty. Research assistants then documented verbal consent for each participant to ensure voluntary enrolment and comprehension of the study.

## Results

A total of 120 providers completed the structured questionnaire, including 58 TBS and 62 FC providers, 60 in each region (Table 1). Compared to the FC group, TBS providers were older (TBS median age 51, IQR 39–60 years vs. FC 34, IQR 30–36 years; *P* < 0.001) and had more years of practice experience caring for injured patients (TBS median 20, IQR 11–28 vs. FC 5, IQR 3–8; *P* < 0.001). A greater percentage of TBS providers were male (TBS 72.4% vs. FC 51.6%, *P* = 0.019). About 75% of TBS providers did not complete secondary school, with about a quarter of TBS respondents indicated they received no formal education. While the FC group included both physicians and nurses providing injury care, specific provider type (i.e., nurse vs. physician) and surgical specialty were not recorded.

**Table 1:** Respondent demographics (*n* = 120).

		TBS, *N* = 58	FC, *N* = 62	
VARIABLE		(*N*, PERCENTAGE)	(*N*, PERCENTAGE)	*P*-VALUE
Region				0.715
	Southwest	30 (51.7)	30 (48.4)	
	Far-North	28 (48.3)	32 (51.6)	
Age (median, IQR)		51 (39, 60) years	34 (30, 36) years	<0.001
Male sex		42 (72.4)	32 (51.6)	0.019
Highest level of education completed				<0.001
	None	14 (24.1)	0	
	Primary	29 (50.0)	0	
	Secondary	11 (19.0)	12 (19.4)	
	University	2 (3.4)	49 (79.0)	
	Unknown/Other	2 (3.4)	1 (1.6)	
Practice in multiple regions		20 (34.5)	0	<0.001
Years caring for injured patients (median, IQR)		20 (11, 28) years	5 (3, 8) years	<0.001
Practice facility				<0.001
	District Hospital	0	7 (11.3)	
	Regional Hospital	0	43 (69.4)	
	Private Hospital	0	3 (4.8)	
	Military Hospital	0	2 (3.2)	
	Home	57 (98.3)	0	
	Other/Missing	1 (1.7)	7 (11.3)	

*TBS = Traditional bone setters, FC = Formal care providers, IQR = Interquartile range.

### Perceptions of TBS providers

**Figure 1:**
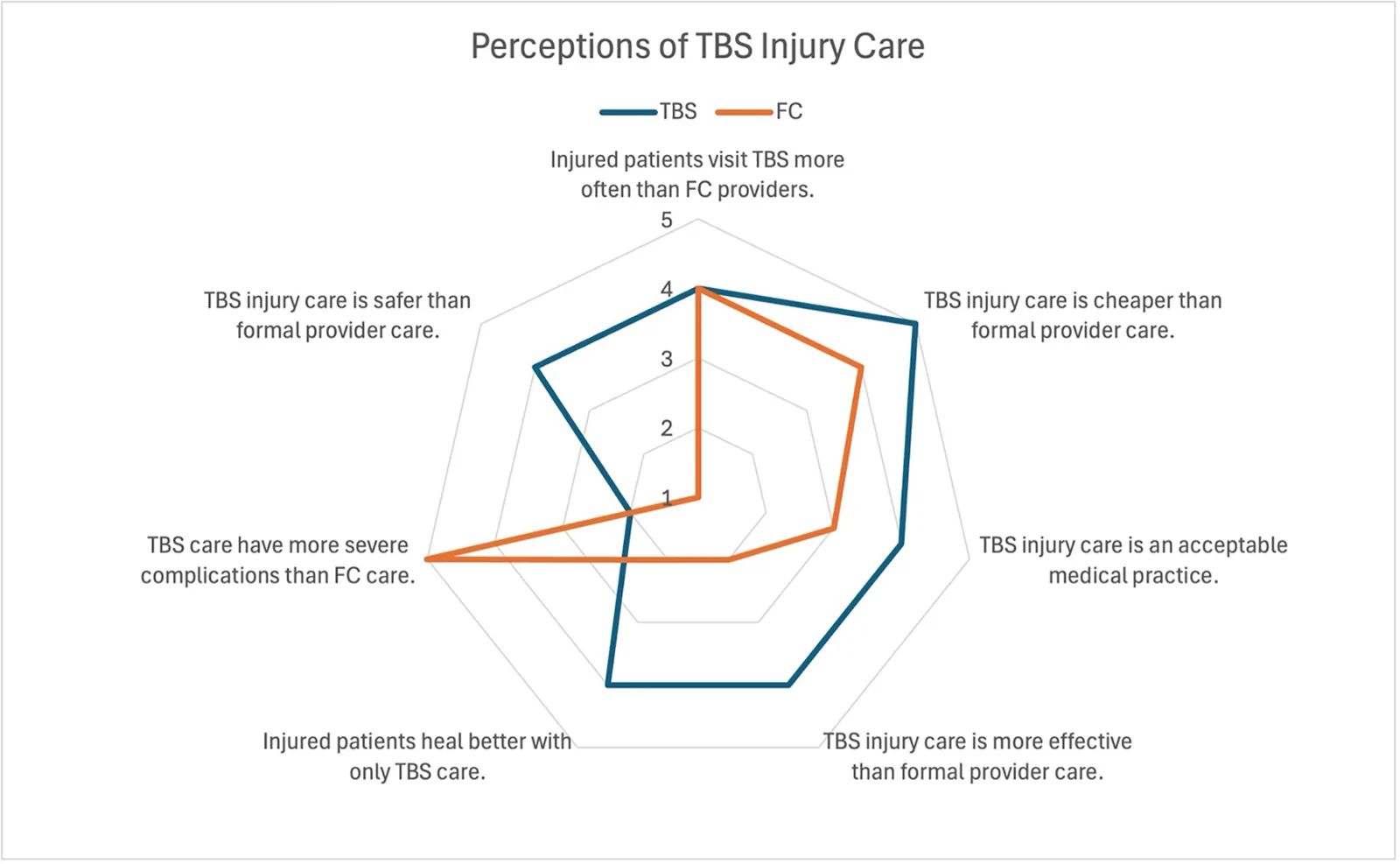
Perceptions of TBS injury care. Radar plot of median Likert scale responses. Legend: 1 = Strongly Disagree, 2 = Disagree, 3 = Neutral, 4 = Agree, 5 = Strongly Agree.

FC and TBS providers differed significantly in their perceptions of TBS care. While TBS providers agreed that their injury care practice was safe, FC providers did not concur (TBS median response “Agree” vs. FC “Strongly Disagree,” *P* < 0.001) (Figure 1). There was also significant discordance regarding perceptions of the efficacy of TBS injury care. TBS providers thought their care to be more effective (TBS “Agree” vs. FC “Disagree,” *P* < 0.001). Moreover, 81% (*n* = 47) of TBS providers agreed with the statement, “Injured patients heal better with only traditional bone setter care,” while 76% (*n* = 47) of FC providers disagreed (*P* < 0.001). There was significant disagreement regarding care complications, as 68% (*n* = 42) of FC providers strongly believed that TBS care had more severe complications, while 64% (*n* = 37) of TBS providers disagreed or strongly disagreed (*P* < 0.001). Both TBS (66%, *n* = 38) and FC (56%, *n* = 35) providers agreed that injured patients seek TBS injury care more often (*P* = 0.19). The groups also believed that TBS care is cheaper compared to FC care (TBS “Strongly Agree” vs. FC “Agree,” *P* = 0.001). Although FC providers remained neutral on whether TBS care is an acceptable practice (TBS “Agree” vs. FC “Neutral,” *P* < 0.001), 24% (*n* = 15) of surveyed FC providers indicated that they personally received care from a TBS provider in their lifetime.

### Perceptions of FC providers

**Figure 2:**
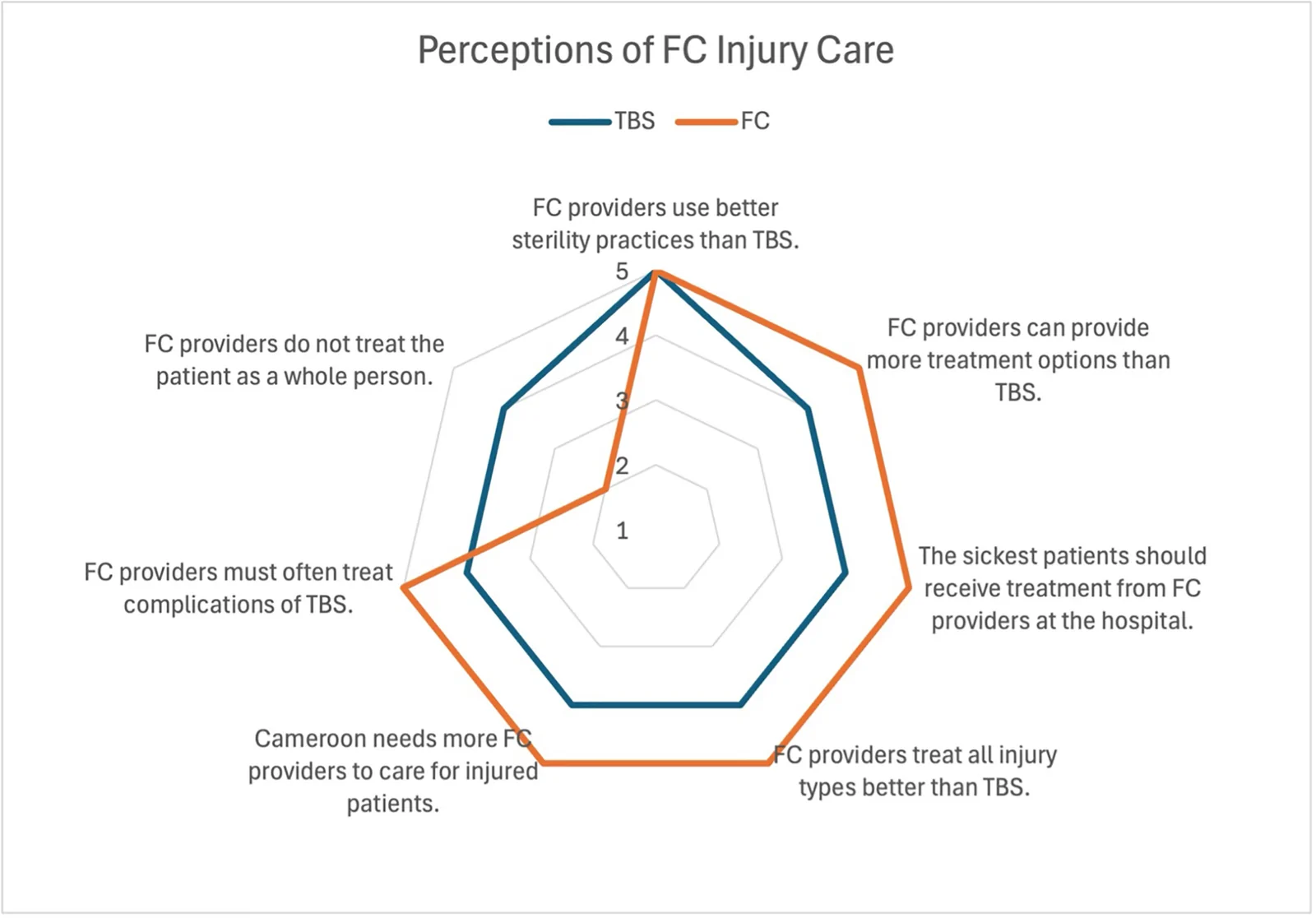
Perceptions of FC injury care. Radar plot of median Likert scale responses. Legend: 1 = Strongly Disagree, 2 = Disagree, 3 = Neutral, 4 = Agree, 5 = Strongly Agree.

When asked about FC injury care, both groups appeared to agree in several instances; however, the FC group often indicated a stronger level of agreement to most statements compared to TBS providers (Figure 2). While TBS agreed that FC can treat all injury types better, provide more treatment options, and should treat the sickest patients, FC providers agreed more strongly (*P* < 0.001). Both groups also strongly agreed that FC providers used better sterility practices. Furthermore, TBS agreed that FC providers often treat complications of TBS injury care (TBS “Agree” vs. FC “Strongly Agree,” *P* < 0.001). TBS also indicated that they believed FC providers did not treat the patient as a whole person (TBS “Agree” vs. FC “Disagree,” *P* = 0.002). Though TBS agreed that Cameroon required more FC providers to treat injured patients, only 36% (*n* = 21) of TBS providers disclosed personally receiving injury care from a FC provider.

### Collaboration between FC and TBS providers

**Figure 3:**
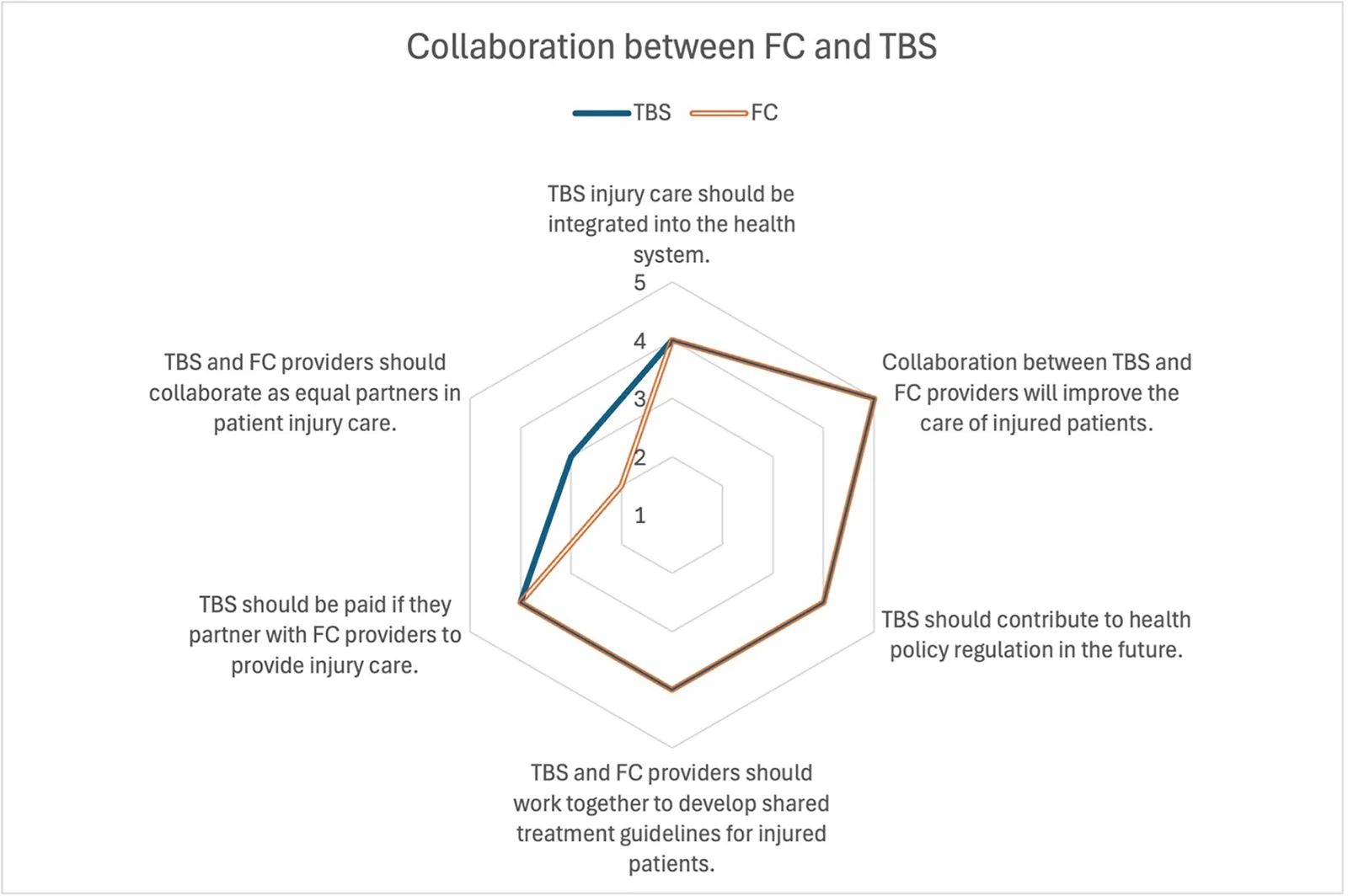
Collaboration between FC and TBS providers. Radar plot of median Likert scale responses. Legend: 1 = Strongly Disagree, 2 = Disagree, 3 = Neutral, 4 = Agree, 5 = Strongly Agree.

The majority of TBS (93%, *n* = 54) and FC providers (77%, *n* = 48) indicated a willingness to collaborate. Both groups strongly believed that collaboration will improve care for injury patients in Cameroon (Figure 3). Providers agreed that TBS should be integrated into the greater health system, contribute toward future health policy, and be compensated for their efforts in such a collaboration. Additionally, both TBS and FC providers agreed that they should develop treatment guidelines for injured patients. Yet, the groups disagreed regarding whether TBS and FC providers should be equal partners in future collaborations (TBS “Neutral” vs. FC “Disagree,” *P* = 0.006).

**Figure 4:**
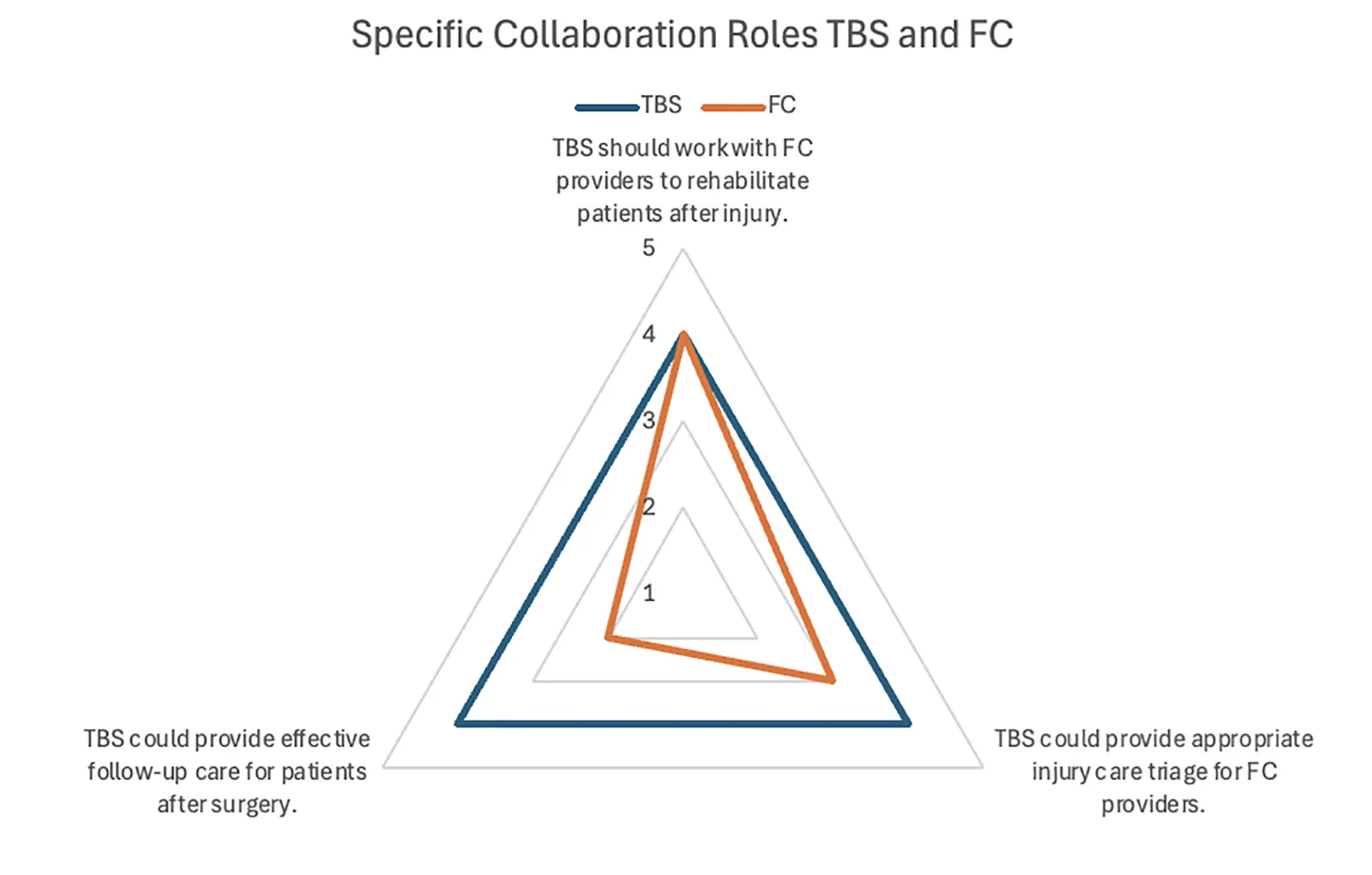
Specific collaborative roles between FC and TBS providers. Radar plot of median Likert scale responses. Legend: 1 = Strongly Disagree, 2 = Disagree, 3 = Neutral, 4 = Agree, 5 = Strongly Agree.

Regarding specific collaborative roles between TBS and FC providers, both groups agreed that TBS can work to rehabilitate injured patients (Figure 4). However, the cohorts differed in their strength of agreement (*P* < 0.001) as the FC group clustered more toward neutral (interquartile range [IQR]: “Neutral”–“Agree”) and the TBS responses skewed more toward a stronger agreement (IQR “Agree”–“Strongly Agree”) (Table 2). However, when assessing if TBS can provide triage care for injured patients, TBS providers agreed, but FC providers remained neutral (*P* < 0.001). Finally, FC providers disagreed that TBS could provide post-operative follow-up care, though TBS providers believed they could provide this type of care (*P* < 0.001).

**Table 2:** Descriptive statistics of survey responses (*n* = 120; TBS *n* = 58, FC *n* = 62).

STATEMENT		MEDIAN	MEAN	IQR	*P*-VALUE
**Perceptions of TBS Injury Care**					
Injured patients visit TBS more often than FC providers	TBS	4, Agree	3.8, Agree	3–5	0.19
	FC	4, Agree	3.4, Neutral	2–5	
TBS injury care is cheaper than formal provider care	TBS	5, Strongly Agree	4.5, Strongly Agree	4–5	0.001
	FC	4, Agree	3.8, Agree	3–5	
TBS injury care is an acceptable medical practice	TBS	4, Agree	3.9, Agree	3–5	<0.001
	FC	3, Neutral	3, Neutral	2–4	
TBS injury care is more effective than FC	TBS	4, Agree	4.1, Agree	4–5	<0.001
	FC	2, Disagree	2.1, Disagree	1–3	
Injured patients heal better with only TBS care	TBS	4, Agree	4.2, Agree	4–5	<0.001
	FC	2, Disagree	2.2, Disagree	1–2	
TBS care have more severe complications than FC care	TBS	2, Disagree	2.5, Neutral	2–3	<0.001
	FC	5, Strongly Agree	4.5, Strongly Agree	4–5	
TBS injury care is safer than formal provider care	TBS	4, Agree	3.9, Agree	4–5	<0.001
	FC	1, Strongly Disagree	1.7, Disagree	1–2	
**Perceptions of FC Injury Care**					
FC providers use better sterility practices than TBS	TBS	5, Strongly Agree	4.4, Agree	4–5	0.001
	FC	5, Strongly Agree	5.0, Strongly Agree	5–5	
FC providers can provide more treatment options than TBS	TBS	4, Agree	4.1, Agree	4–5	0.001
	FC	5, Strongly Agree	4.7, Strongly Agree	5–5	
The sickest patients should receive treatment from FC at the hospital	TBS	4, Agree	3.9, Agree	4–5	<0.001
	FC	5, Strongly Agree	4.7, Strongly Agree	5–5	
FC providers treat all injury types better than TBS	TBS	4, Agree	3.9, Agree	3–5	0.002
	FC	5, Strongly Agree	4.7, Strongly Agree	5–5	
Cameroon needs more FC to care for injured patients	TBS	4, Agree	4.2, Agree	4–5	<0.001
	FC	5, Strongly Agree	4.9, Strongly Agree	5–5	
FC providers must often treat complications of TBS	TBS	4, Agree	3.5, Neutral	3–5	<0.001
	FC	5, Strongly Agree	4.5, Strongly Agree	4–5	
FC providers do not treat the patient as a whole person	TBS	4, Agree	3.2, Neutral	2–4	0.002
	FC	2, Disagree	2.5, Neutral	2–4	
**Collaboration between FC and TBS Providers**					
TBS injury care should be integrated into the health system	TBS	4, Agree	4.2, Agree	4–5	0.07
	FC	4, Agree	3.8, Agree	3–5	
TBS and FC collaboration will improve care of injured patients	TBS	5, Strongly Agree	4.7, Strongly Agree	4–5	0.06
	FC	5, Strongly Agree	4.3, Agree	4–5	
TBS should contribute to health policy regulation in the future	TBS	4, Agree	3.8, Agree	3–4	0.58
	FC	4, Agree	3.5, Neutral	2–5	
TBS and FC should work together to develop shared treatment guidelines for injured patients	TBS	4, Agree	3.6, Agree	3–4	0.94
	FC	4, Agree	3.5, Neutral	3–4	
TBS should be paid if they partner with FC to provide injury care	TBS	4, Agree	3.7, Agree	3–5	0.91
	FC	4, Agree	3.9, Agree	3–5	
TBS and FC should collaborate as equal partners in patient injury care	TBS	3, Neutral	2.8, Neutral	2–4	0.006
	FC	2, Disagree	2.1, Disagree	1–3	
TBS should work with FC to rehabilitate patients after injury	TBS	4, Agree	4.2, Agree	4–5	<0.001
	FC	4, Agree	3.5, Neutral	3–4	
TBS could provide appropriate injury care triage for FC	TBS	4, Agree	4.2, Agree	4–5	<0.001
	FC	2.5, Neutral	2.8, Neutral	2–4	
TBS could provide effective follow-up care for patients after surgery	TBS	4, Agree	3.7, Agree	4–4	<0.001
	FC	2, Disagree	2.1, Disagree	1–3	

*TBS = Traditional bone setters, FC = Formal care providers, IQR = Interquartile range.

**Numeric categorization of responses: Strongly Disagree = 1–1.5, Disagree = 1.6–2.4, Neutral = 2.5–3.5, Agree = 3.6–4.4, and Strongly Agree = 4.5–5.

## Discussion

Our study identified a shared willingness to collaborate between the TBS and FC cohorts in several key domains. Both groups believed that collaboration will ultimately benefit injured patients and TBS providers should be included in future health policy discussions. Additionally, there was agreement that the two cohorts should work together to draft treatment guidelines for injured patients. Notably, both TBS and FC providers expressed a desire to collaborate on injury rehabilitation, which could be a crucial area for initial collaborative efforts. A prior Cameroonian study reported that patients sometimes resort to TBS methods after undergoing the prescribed FC treatment in a bid to boost or accelerate healing, possibly reflecting a role of traditional care in rehabilitation [10]. Despite this alignment, important differences emerged regarding the scope of collaboration. For example, there was disagreement regarding TBS providers’ ability to conduct triage and post-operative follow-up care. This discordance may stem from contrasting perceptions of TBS competence among the cohorts and may represent potential barriers to effective future collaboration.

Although traditional healers have been studied across sub-Saharan Africa, there remains a relative lack of research focused specifically on TBS in Cameroon, particularly from the perspective of FC providers [6, 8, 10, 15]. To our knowledge, this is one of the first studies that formally surveys opinions of FC providers toward TBS care in Cameroon. Understanding provider attitudes toward partnership will facilitate future collaboration between TBS and FC providers to increase patient access to injury care. The recent passage of Cameroonian Law No. 2024/018 provides a legal framework for such integration. Our study findings can serve as a foundational evidence base to eventually integrate TBS care within the larger Cameroonian health system over time.

TBS and FC providers’ desire to collaborate aligns with prior African studies that demonstrate traditional healers’ aspiration to collaborate with the formal health care system [9, 16]. However, FC providers in our study expressed skepticism regarding the safety and efficacy of TBS care, a perspective that may be shaped by their clinical experience. These providers often encounter patients only after complications have occurred, such as delayed treatment or improperly managed injuries, which may introduce a bias in FC perception of TBS practice. As a result, FC providers may predominantly see the adverse outcomes of TBS care rather than its potential benefits in less severe or successfully managed cases [7, 14, 20, 21]. The predominantly rural, under-resourced demographics of our two study regions may further sharpen this dynamic, since formal injury services are scarcest precisely where TBS are most heavily relied upon; perceptions formed in such settings may not generalize to more urbanized regions. Despite these negative perceptions, FC providers in this study indicated a desire to work with TBS providers. These findings are consistent with prior Nigerian focus group data that revealed FC providers’ negative opinions toward TBS care but tacitly supported the integration of TBS into the FC system with proper training [22].

There are several limitations in this study. First, data collection was conducted in only two regions of Cameroon. The perceptions and practices of TBS and FC providers may differ in other regions throughout the country, limiting the generalizability of our findings. While the sample size was sufficient for the statistical analysis conducted, it may not fully capture the diversity of opinions within each provider group. Moreover, though snowball sampling remained the optimal method for enrolling TBS participants, who were difficult to identify due to a lack of formal registries, this method can lead to conformity bias. Collegial referral chains tend to link providers who share professional networks and viewpoints, resulting in group homogeneity. Additionally, data were collected shortly after the passage of Cameroonian Law No. 2024/018. Ongoing efforts to formalize TBS integration during this period may have begun to shape provider attitudes, further limiting comparability of these findings with settings where such efforts are absent. Finally, while using a structured questionnaire with a Likert scale facilitates ease of data analysis, other research methods like qualitative interviews may provide richer, more nuanced insights into each cohort’s perceptions.

Future interventions should prioritize collaborative models that build on the areas of agreement identified in this study, particularly rehabilitation and the development of treatment guidelines as initial entry points for integration between TBS and FC providers. Any intervention should leverage the presence and cultural acceptance of TBS while ensuring patient safety. For instance, FC and TBS providers can jointly develop care guidelines for managing common injuries such as open fractures. These guidelines could initially focus on areas of high agreement and establish shared protocols for injury care. As collaboration strengthens and mutual trust grows, additional responsibilities for TBS, such as basic triage or referral initiation, may be considered to increase escalation to FC providers for severe or complex injuries requiring a higher level of care. While allopathic pedagogy from FC providers would heavily influence treatment guidelines, TBS could instruct FC providers in patient-centered communication approaches that Cameroonian patients prefer [23]. Likewise, an intervention studying the impact of TBS providers in post-injury rehabilitation would likely garner support from both cohorts.

## Conclusion

FC and TBS providers wish to collaborate to expand injury care for injured patients in Cameroon. While both groups agree that TBS may be able to provide rehabilitation for injured patients, there remains significant disagreement regarding other types of care such as triage and post-operative follow-up. The utilization of TBS within the larger health care system in Cameroon can potentially expand care injury access and improve trauma outcomes.

## Data Availability

The de-identified data supporting the findings of this study are available from the corresponding author upon reasonable request.
